# Spatially targeting *Culex quinquefasciatus *aquatic habitats on modified land cover for implementing an Integrated Vector Management (IVM) program in three villages within the Mwea Rice Scheme, Kenya

**DOI:** 10.1186/1476-072X-5-18

**Published:** 2006-05-09

**Authors:** Benjamin G Jacob, Josephat Shililu, Ephantus J Muturi, Joseph M Mwangangi, Simon M Muriu, Jose Funes, John Githure, James L Regens, Robert J Novak

**Affiliations:** 1 Illinois Natural History Survey, Center for Ecological Entomology, 607 East Peabody Dr. Champaign IL 61820, USA; 2 Human Health Division, International Centre of Insect Physiology and Ecology (ICIPE)), Nairobi, Kenya; 3 Department of Occupational and Environmental Health, University of Oklahoma Health Sciences Center, 801 N.E. 13^th ^Street, Oklahoma City, OK 73104, USA

## Abstract

**Background:**

Continuous land cover modification is an important part of spatial epidemiology because it can help identify environmental factors and *Culex *mosquitoes associated with arbovirus transmission and thus guide control intervention. The aim of this study was to determine whether remotely sensed data could be used to identify rice-related *Culex quinquefasciatus *breeding habitats in three rice-villages within the Mwea Rice Scheme, Kenya. We examined whether a land use land cover (LULC) classification based on two scenes, IKONOS at 4 m and Landsat Thematic Mapper at 30 m could be used to map different land uses and rice planted at different times (cohorts), and to infer which LULC change were correlated to high density *Cx. quinquefasciatus *aquatic habitats. We performed a maximum likelihood unsupervised classification in Erdas *Imagine *V8.7^® ^and generated three land cover classifications, rice field, fallow and built environment. Differentially corrected global positioning systems (DGPS) ground coordinates of *Cx. quinquefasciatus *aquatic habitats were overlaid onto the LULC maps generated in ArcInfo 9.1^®^. Grid cells were stratified by levels of irrigation (well-irrigated and poorly-irrigated) and varied according to size of the paddy.

**Results:**

Total LULC change between 1988–2005 was 42.1 % in Kangichiri, 52.8 % in Kiuria and and 50.6 % Rurumi. The most frequent LULC changes was rice field to fallow and fallow to rice field. The proportion of aquatic habitats positive for *Culex *larvae in LULC change sites was 77.5% in Kangichiri, 72.9% in Kiuria and 73.7% in Rurumi. Poorly – irrigated grid cells displayed 63.3% of aquatic habitats among all LULC change sites.

**Conclusion:**

We demonstrate that optical remote sensing can identify rice cultivation LULC sites associated with high *Culex *oviposition. We argue that the regions of higher *Culex *abundance based on oviposition surveillance sites reflect underlying differences in abundance of larval habitats which is where limited control resources could be concentrated to reduce vector larval abundance.

## Background

The "focal" nature of arboviruses suggests that there are inherent landscape factors underlying the timing and distribution of transmission [1]. Human-induced land use land cover (LULC) changes are the primary drivers of a range of arbovirus disease outbreaks and emergence events and also modifiers of the transmission of endemic infections [2]. LULC changes in a rice-village ecosystem include road construction, wetland modification, agricultural encroachment, dam building, irrigation, and other activities. These changes in turn cause a cascade of factors that exacerbate infectious diseases in African ricelands caused by *Culex *mosquitoes [3] such as West Nile Virus (WVN), Rift Valley Fever (RVF), Yellow fever and *Bancroftian filariasis*[4]. For example, an increase in soil moisture associated with irrigation development in the southern Nile Delta, following the construction of the Aswan High Dam, caused a rapid rise in *Cx. quinquefasciatus*, and consequential increase in the arthropod-borne disease, Bancroftian filariasis [5,6]. In settlements of the Kenya Coast, *Cx. pipens *was found to be a very potent vector of *Wuchereria bancrofti *filariasis which developed in three major types of urban modified land cover namely; pit latrines, cesspools and small cement cisterns [7]. An isolate of West Nile Virus (WNV) was obtained from a pool of four male *Culex *mosquitoes while conducting an investigation of Rift Valley Fever (RVF) in the Turkwel Gorge Hydroelectric Project within an irrigational compound along the Kenya-Uganda border [8]. A recently constructed paved road linking agricultural towns Kabarnet with Eldoret to the west and Marigat to the East in the Great Rift valley in Kenya recorded an outbreak of Yellow fever in 1992 [9].

Increasingly in recent years, geographic information systems (GIS) and remote sensing data has been used to model the spatial and seasonal dynamics of diseases involving invertebrates as intermediate hosts [10,11], and to develop affordable early warning systems [12]. GIS/remote sensing data are able to discriminate several factors important to mosquito production, including estimation of rice planted area [13-18] for different rice cultural practices and planting dates [15,19]. Immature collection of mosquito larval data can be incorporated into a GIS along with satellite imagery, and classified to land use to estimate abundance and distribution of mosquito larval habitats [20].

The initial objective of our study was to determine whether spatial variation in abundance of *Cx. quinquefasciatus *aquatic habitat in three East African village complexes were related to remotely measured changes in LULC classified as built environment, rice field and fallow. In order to evaluate the efficiency of GIS/remote sensing in identifying *Cx. quinquefasciatus *as measured by oviposition surveillance sites, we examined whether 1988 Landsat TM at 30 m spatial resolution and 2005 IKONOS data at 4 m spatial resolution can be used to map man-made LULC change over a period of 17 year in three East African rice villages. Landsat TM at 30 m have been used to estimate the areas of paddy rice fields [14,16,21,22] and have been used to characterize immature habitats of flood-water mosquitoes [23]. IKONOS 4 m-multispectral imagery allows the relative contributions of mosquito larval habitats in a rice field to be determined and their abundance to be mapped [24]. By relating *Culex *abundance from fixed surveillance sites to the underlying landscape configuration, areas of high risk to incidental mammalian hosts may be identified and more effective mosquito control employed.

## Methods

### Study sites

The studies were conducted 100 km North East of Nairobi, in Kangichiri, Kiuria and Rurumi villages within the Mwea Rice Scheme, Kenya. Mwea occupies the lower altitude zone of Kirinyaga District in an expansive low-lying formally wet-savannah ecosystem. The Mwea rice irrigation scheme is located in the west central region of Mwea Division and covers an area of about 13,640 ha. More than 50% of the scheme area is used for rice cultivation. The remaining area is used for subsistence farming, grazing and community activities or remains close to its original state. The mean annual precipitation is 950 mm with maximum rainfall occurring in April/May and October/November. The average temperatures range from 16–26.5°C. Kangichiri and Kiuria are located at the central-west region of the scheme. Rurumi is located in the north west of the scheme. The number of homesteads in Kiuria, Kangichiri and Rurumi is 222, 231 and 262, respectively. Each village has approximately 650 residents. Many species of birds and mammals visit the rice fields for feeding from surrounding areas and are generally considered as temporary or ephemeral inhabitants. Birds and mammals can act as intermediary hosts for arboviruses transmission [4].

### Larvae sampling

Larval sampling was conducted in 193, 190 and 193 randomly selected aquatic habitats in Kangichiri, Kiuria and Rurumi, respectively. The habitats were classified as temporary, semi-permanent or permanent depending on the period they were capable of holding water. Habitats were defined as temporary if they held water for upto 2 weeks, semi-permanent if they sustained water for up to 3 months or permanent if they held water for a period equal to or longer than 6 months. All water bodies were inspected for mosquito larvae using standard dipping techniques with a 350-ml dipper to collect the mosquito larvae and pupae [25]. The mosquitoes were temporarily held in plastic bags and transported to the laboratory where they were sorted by sub family, counted and recorded. *Culex *mosquito larvae were identified morphologically to species using taxonomic keys at the Human Health Division, International Centre of Insect Physiology and Ecology (ICIPE) in Nairobi, Kenya.

Each *Culex *larval habitat with its associated land cover attributes from each study site was entered into a Vector Control Management System (VCMS) (Advanced Computer resources Corp (ACR), 100 Perimeter Road, Nashua, NH, USA) database. The VCMS plotted and updated all differentially corrected global positioning systems (DGPS) ground coordinates from a CSI -Wireless Max receiver used to acquire ground coordinates of each aquatic habitat. The VCMS exported the data to a spatial format for use in GIS and for generating LULC maps.

### Satellite data

The satellite information obtained from IKONOS was obtained in June 2005 and encompassed visible and near-infra-red (NIR) wavebands 2(0.45–0.52 μm), 3 (0.52–0.60 μm), 4 (0.63–0.69 μm) and 5(0.76–0.90 μm). This information was used to quantify all *Cx. quinquefasciatus *aquatic habitats in the three study sites. The information obtained from the TM included bands 1 (0.45–052 μm), 2 (0.52–0.60 μm), 3(0.63–0.69 μm) and 4(0.76–0.9.0 μm) and was obtained from Landsat 5 in June 1988. Information from Landsat TM channels 1, 2, 3, 4 can distinguish between high and low mosquito producing rice fields [26]. The spectral characteristics of the IKONOS multi-spectral bands are approximately the same as the Landsat TM bands 1 through 4 [27]. The geographic projection used for all of the spatial datasets is the Universal Transverse Mercator (UTM) Zone 37S datum WGS-84 projection.

**Figure 1:**
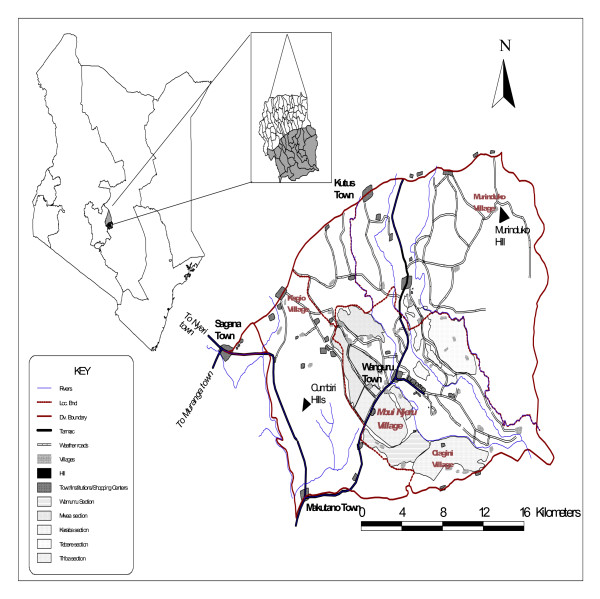
**Base map of the Mwea rice scheme, Kenya**. This map shows the location of Mwea irrigation scheme in relation to the other parts of the country. In addition, it also shows the area covered by the Scheme.

The IKONOS and TM images were registered based on the position of the sensors when the images were generated. We geo-registered the remaining datasets, which involved aligning known control-point locations such as cross roads and hydrological bodies with exactly the same locations stored in the datasets. The referenced coordinates of the control points were obtained from existing base maps that were created from previous ground surveys of the Mwea rice scheme (Figure 1). ArcInfo 9.1^® ^adjusted the datasets so that the control point locations, whose coordinates were entered into the spatial dataset, were correctly positioned relative to each other.

### Spatial datasets

Land cover was determined from each of the images using ERDAS *Imagine *V8.7^® ^[28] Datasets created for Kangichiri, Kiuria and Rurumi study sites included three LULC classifications: rice field, built environment, and fallow cover classes.

1) Built environment: comprised of areas of intensive use with much of the land covered by physical infra structures. This land cover also included homesteads, holding areas for livestock such as corrals, farm lanes and roads, ditches and canals (irrigation infrastructure).

2) Fallow: Paddies without canopies e.g. transplant early tiller stage with little canopy covering water.

3) Rice field: Paddies where the vegetative growth shades the water and or ground.

The change in LULC change that occurred between 1988–2005 were classified into the following classes: rice field to built environment, fallow to built environment, rice field to fallow, built environment to fallow. Pixels that could not be classified were categorized as maintained built environment, maintained fallow or maintained rice field. The spatial distribution of the larval mosquito collections was overlaid on the land-use image derived in ArcInfo 9.1^®^, and the number of mosquito aquatic habitats in each class was calculated.

#### Data analysis strategy

A digitized custom grid tracing each paddy was generated in Arc Info 9.1^® ^[29]. This provided for a unique identifier which was placed in each grid cell (= paddy). The grid extended out to a 1 kilometer area from the external boundary of each village providing a 1 km radial area. Stratifying the grid involved assessing the level of irrigation in each grid cell and assigning a value of 1 if the grid cell was well-irrigated and 0 if the paddy was poorly-irrigated. A grid cell was classified as rice well-irrigated if there was appropriate drainage system, clear of debris, were present with no standing water visible or it was located on a slope providing gravity driven irrigation. Rice fields were classified as poorly-irrigated if irrigation systems had no functional drainage systems or were in dead-end locations such as depressions or valleys. The grid was also characterized by distance between houses, road types, graded, gravel, foot paths including those between villages, village to paddy community water sources, and access to utilities. Information contained in the 1999 Kenya National census and District Development Report, environmental descriptions from field surveys and topographical maps were used to assist with the stratification process. The boundaries of selected grid cells were located in the field using hand-held navigational units from DGPS and base-maps with permanent extent of grid cell boundaries. Twenty-five grid cells were selected from each stratum (*n *= 50). A systematic random sample with a random start was used to select rice paddies. This ensured that the probability of selection was equal for each grid cell within the respective strata. We overlaid the sampling unit grid with the larval spatial datasets to identify the LULC pixels within each grid cell of interest. All potential aquatic larval habitat sites were identified, and data relative to species composition and abundance, predators, water quality and environmental parameters were collected longitudinally. The entomological variable was total *Culex *larvae present.

We performed an independent sample t test to compare the differences in *Culex *mosquito density between the well and the poorly drained strata. We examined the LULC for each sample unit at the study sites to determine the amount of the land cover that changed between 1988 and 2005. A multivariate analysis of variance (MANOVA) was performed to adequately control for unobserved heterogeneity as well as factors known to influence mosquito larval ecology such as canopy, water depth, rice height and tillers. All data management and calculations were performed using SPSS 11.5^® ^[30].

## Results

Overlaying a grid on high resolution data can help organize and characterize mosquito larval habitats in relation to ecological attributes about an aquatic larval habitat and community-level drainage potential and investigate whether agricultural activities within or around a urban ecosystem will increase the probability of disease occurrence [31]. In Kangichiri, a total of 193 habitats belonging to four habitat types namely paddies (54.1%), canals (45.9%), pools (1.1%), and marshes (0.8%) were sampled. Majority of these habitats were poorly drained (57.1%). The difference in the density of immature *Culex *was not significant between the well and poorly drained strata (t = 0.28, df = 1, 192, p = 0.60). In Kiuria, a total of 191 habitats belonging to three habitat types namely paddies (62.6%), canals (37.4 %) and pools (1.6%) were identified. Majority of these habitats were poorly drained (50.7%), and had significantly higher *Culex *mosquito density compared to the well drained strata (t = 6.65 df = 1, 190, p = 0.01). In Rurumi, a total of 193 habitats belonging to three habitat types namely paddies (52.3%), canals (47.3%) and pools (0.42%) were sampled. Majority of these habitats were poorly drained (51.9%). The difference in the density of immature *Culex *was not significant between the well and the poorly drained strata (t = 2.47, df = 1, 192, p = 0.12).

**Figure 2:**
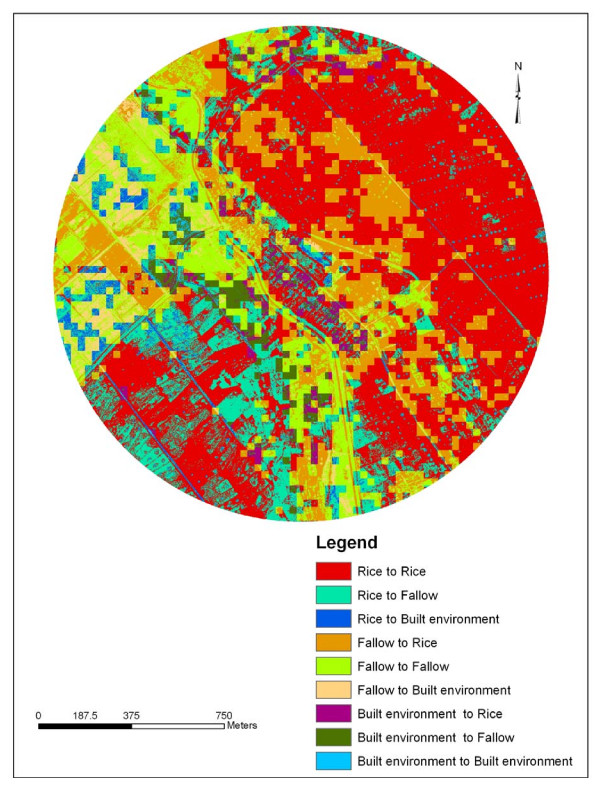
**Land use land cover change for Kangichiri study site between June 1988 and June 2005**. This map shows the areas of change and non change that has taken place in Kangichiri study site over a 17-year period between 1988 and 2005. The figure was generated using IKONOS and Landsat TM.

**Figure 3:**
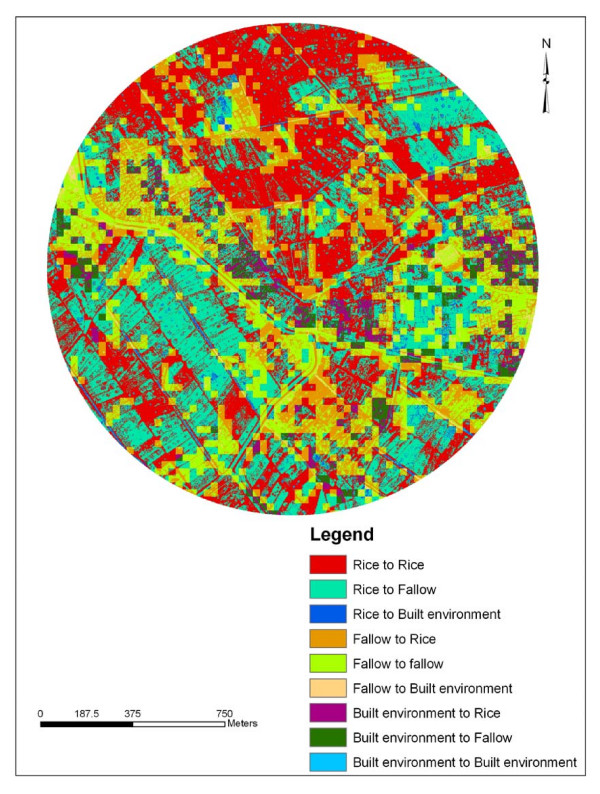
**Land use land cover change for Kiuria study site between June 1988 and June 2005**. This map shows the areas of change and non change that has taken place in Kiuria study site over a 17-year period between 1988 and 2005. The figure was generated using IKONOS and Landsat TM.

**Figure 4:**
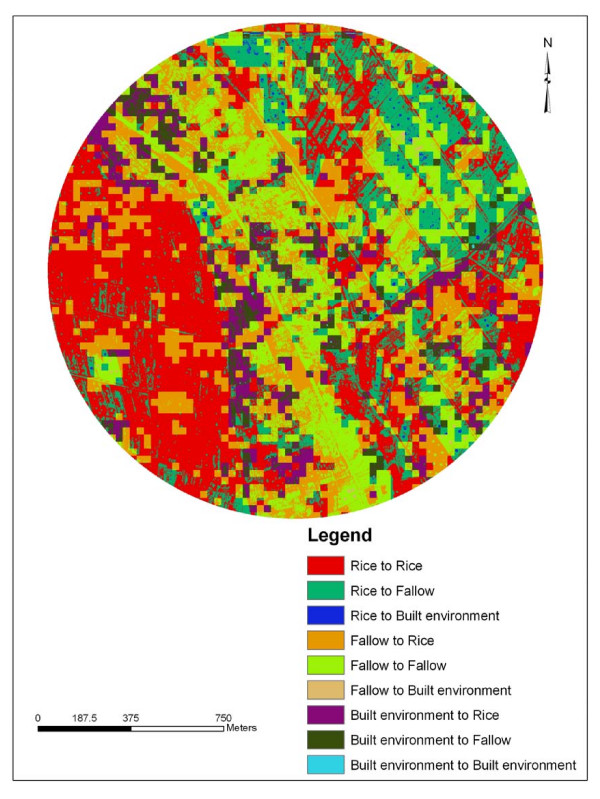
**Land use land cover change for Rurumi study site between June 1988 and June 2005**. This figure shows the areas of change and non change that has taken place in Rurumi study site over a 17-year period between 1988 and 2005. The figure was generated using IKONOS and Landsat TM

Rapid screening and identification of larval habitats on modified land cover sites is a key element in designing and implementing vector control programs [11] GIS maps can help organize and characterize mosquito larval habitats in relation to LULC attributes [32]. Land cover changes between 1988 and 2005 were analyzed in ArcInfo 9.1^® ^and mapped. The percentage of overall LULC change for the 17-year period was 42.1 % in Kangichiri, 52.8 % in Kiuria and 50.6% in Rurumi (Table 1). The most common LULC change was fallow to rice field in Kangichiri (Figure 2), rice field to fallow in Kiuria (Figure 3) and fallow to rice field in Rurumi (Figure 4). The proportion of aquatic habitats positive for *Culex *larvae in LULC change sites was 77.5% in Kangichiri, 72.9% in Kiuria and 73.7% in Rurumi (Table 2). The most frequent LULC change positive for *Culex *larvae was fallow to rice field in Kangichiri, rice field to fallow in Kiuria and fallow to rice field in Rurumi (Table 3). In all three study sites, there was a significant difference in LULC change and the number of aquatic habitats positive for *Culex *larvae (F = 2.27 df = 5,194, p = 0.05). The MANOVA model used to examine the effects of depth, canopy and LULC change accounted for 57% of the variation of *Culex *density.

**Table 1:** Proportion of overall land cover change over 17 years in Kangichiri, Kiuria and Rurumi. This table is a summary of the overall land use land cover change that has taken place in the three study sites namely; Kangichiri, Kiuria and Rurumi between June 1988 and June 2005

Village	Total area in square kilometers	Total area in square Kilometer of land cover change	Percent Land cover change
Kangichiri	46.3	19.5	42.1
Kiuria	58.2	30.7	52.8
Rurumi	43.5	22.0	50.6

**Table 2:** Number of *Cx. quinquefasciatus *larvae collected in areas of different land cover change. This table summarizes the number of *Cx. quinquefasciatus *larvae collected in different habitat types located within the well and the poorly irrigated strata relative to the type of land use land cover change that have occurred.

Village	Strata	Habitat type	Non LULC Change	Rice field to built environment	Fallow to built environment	Rice field to fallow	Built environment to fallow	Built environment to rice field	Fallow to rice field
Kangichiri	Well irrigated	Paddy	21	5	5	8	8	4	10
		Canal	14	6	10	7	5	6	7
		Pool	0	0	0	0	0	1	0
		
		Total	35	11	15	15	13	11	17
	
	Poorly irrigated	Paddy	23	8	11	13	9	5	14
		Canal	18	7	5	5	8	7	8
		Pool	0	0	0	0	0	0	2
		Marsh	0	0	0	0	0	0	2
		
		Total	41	15	16	18	17	12	26

Kiuria	Well irrigated	Paddy	16	13	18	23	13	7	8
		Canal	7	3	2	22	2	2	4
		
		Total	23	16	20	25	15	9	12
	
	Poorly irrigated	Paddy	22	5	8	9	7	4	4
		Canal	7	7	6	8	8	7	5
		
		Total	69	12	14	17	15	11	9

Rurumi	Well irrigated	Paddy	23	8	14	5	9	7	8
		Canal	11	5	3	7	5	5	4
		Pool	0	0	0	1	0	0	0
		
		Total	34	13	17	13	14	12	22
	
	Poorly irrigated	Paddy	33	3	4	5	1	3	12
		Canal	9	7	10	9	4	9	15
		
		Total	62	10	14	14	5	12	27

**Table 3:** Proportion of aquatic habitats positive for *Cx. quinquefasciatus *in different LULC sites. This table summarizes the proportion of aquatic habitats positive for *Cx. quinquefasciatus *larvae in relation to the land use land cover change that has occurred

Village	LULC change	Number of LULC Habitats	Percent positive for *Culex *larvae
Kangichiri	Rice field to Fallow	33	17.7%
	Fallow to rice field	43	23.1%
	Built environment to fallow	30	16.1%
	Fallow to built environment	31	16.7%
	Built environment to rice field	23	12.4%
	Rice field to built environment	26	14.0%
	
	Total	186	100

Kiuria	Rice field to Fallow	42	24.0%
	Fallow to rice field	21	12.0%
	Built environment to fallow	30	17.1%
	Fallow to built environment	34	19.4%
	Built environment to rice field	20	11.4%
	Rice field to built environment	28	16.0%
	
	Total	175	100

Rurumi	Rice field to Fallow	27	15.6%
	Fallow to rice field	49	28.3%
	Built environment to fallow	19	11.0%
	Fallow to built environment	31	17.9%
	Built environment to rice field	24	13.9%
	Rice field to built environment	23	13.3%
	
	Total	173	100

## Discussion

The relative abundance of larval habitats positive for *Culex *larvae were higher in fallow to rice field and rice field to fallow LULC change sites in all study sites. The higher preponderance of rice cultivation LULC's can influence arbovirus disease transmission in several ways. Rice farming activities (e.g., excavation, building construction, and irrigation schemes), and increases in human activity can increase opportunities for *Culex *mosquitoes through the enhancement of shallow bodies of water and through an increase in the number of artificial water collection reservoirs. Small, flooded depressions in the soil, tire tracks and shallow ditches are common aquatic habitats for *Cx. quinquefasciatus *[3]. Rice agriculture can influence adult abundance due to the abundance of larval habitats such as catch basins, numerous open containers of varying dimensions that hold water and organic material long enough for development of larvae to adult stage. Rice environments can also provide ample aquatic habitats for mosquitoes through physical deterioration of hydrological networks (e.g., broken or blocked water drains, potholes, and rubbish). Larvae of the *Cx quinquefasciatus *complex (*Cx. quinquefasciatus *and *Cx. torrentium*) are abundant in highly polluted pools [33]. Many species of *Culex *mosquitoes prefer to oviposit in organically polluted or eutrophic aquatic sites [34].

Drained paddies which had subsequently filled with rainwater and flood water were found to be the most important habitat in all three study sites. The importance of proper drainage should be stressed as one tool for reducing *Culex *aquatic habitats. Poor water husbandry can result in prolific breeding of culicine mosquitoes [35]. Current modification projects should involve the creation of channels to improve water flow in areas of standing water, filling small ponds or water-collecting depressions, or changing the banks of water impoundments to reduce mosquito populations in all rice cultivation LULC change sites. Since canals can create *Culex *larval breeding sites, particularly in slow-moving pools with heavy vegetation, re-grading streams and even straightening canals may reduce aquatic habitats. Water flow > 20 cm/sec is considered necessary for prevention of *Cx. quinquefasciatus *breeding [36]. Alternatively, engineering to prevent waste water discharge into open channels may improve water quality, leading to mosquito suppression through colonization of larvivorous predators.

An unsupervised algorithm using 2005 IKONOS data with TM data from 1988 in ArcInfo 9.1^® ^provided informative LULC change data for *Cx. quinquefasciatus *habitat suitability in Kangichiri, Kiuria and Rurumi. However, one of the most important considerations of satellite data is the increased error in geo-referencing on a pixel-by-pixel basis. ArcInfo 9.1^® ^overlay operations involving adding and ratioing map values which require application of the operation to each pixel; in turn, the problem of error propagation such as location errors through the use of these operations may be relevant to ArcInfo 9.1^® ^[37]. The presence of location error interacting with the spatial structure in the source maps, the presence of spatial correlation in the errors of the attribute measurement process, or indeed their simultaneous presence are capable of generating spatially complex maps of propagated error [37,38]. Inadequate geographic registration could have resulted in misclassification and subsequent underestimation or overestimation of the extent of LULC change in the four study sites. Each scene was co-registered to matching scene and the maximum likelihood algorithm used the pixel classification on all the satellite data. However, the bands within Landsat TM may have failed to capture all spatial and temporal topographic cover. As such, the actual relationship between LULC change and mosquito larval habitats in Kangichiri, Kiuria and Rurumi deserves further clarification through continued field ecologically based research and satellite data.

## Conclusion

The 42.1 % of LULC change for Kangichiri, 52.8 % for Kiuria and 50.6% for Rurumi in the 17-year period contributed to changes in abundance, and distribution of *Cx. quinquefasciatus *larval habitats. There was higher preponderance of aquatic habitats positive for *Culex *larvae in LULC change sites than non-LULC changes in the three study sites. In areas in which LULC change was detected, the highest overall percent in the three study sites positive for *Culex *larvae was fallow to rice field and rice field to fallow. Spatially targeting high density *Cx. quinquefasciatus *aquatic habitats on LULC rice cultivation change sites can develop and implement an integrated vector management (IVM) program based on larval habitat productivity. Treatments or habitat perturbations should be based on surveillance of larvae in the most productive areas of the agro-ecosystem and adjacent village [39]. Unchecked land cover modification and use of inadequate insecticides have promoted wide spreading and proliferation of *Cx. quinquefasciatus *[40]. The impact of larval control using new formulations of insecticides should be rigorously tested using a modified longitudinally designed study on all rice cultivation LULC change sites in Kangichiri, Kiuria and Rurumi. If larval management targeting LULC change sites continues to reduce larval populations in Kangichiri, Kiuria and Rurumi, this program should be expanded to other rice irrigation complexes with a focus on remote and field technology-transfer.

## Competing interests

The author(s) declare that they have no competing interests.

## Authors' contributions

BGJ, helped conceive the study and led the drafting of this manuscript; EJM, JMM and SMM helped collect and analyze the data; JF did the ArcInfo 9.1^® ^overlay operations and generated the satellite maps; JS supervised the field data collection and helped analyze the data; JG, JLR jointly conceived and designed the study; RJ is the principal investigator of the study. All authors interpreted the results and wrote the paper.

## References

[B1] Kunkel KE, Novak RJ, Lampman RL, Gu W (2006). Modeling the impact of variable climatic factors on the crossover of *Culex restuans *and *Culex pipiens *(Diptera: Culicidae), vectors of West Nile virus in Illinois. American Journal of Tropical Medicine and Hygiene.

[B2] Patz JA, Daszak P, Tabor GM, Aguirre AA, Pearl M, Epstein J, Wolfe ND, Kilpatrick AM, Foufopoulos J, Molyneux D, Bradley DJ (2004). Unhealthy landscapes: Policy recommendations on land use change and infectious disease emergence. Environmental Health Perspective.

[B3] Mutero CM, Ng'ang'a PN, Wekoyela P, Githure J, Konradsen F (2004). Ammonium sulphate fertilizer increases larval populations of *Anopheles arabiensis *and culicine mosquitoes in rice fields. Acta Tropica.

[B4] Gillett JD, Smith JG (1972). Common African Mosquitoes and their medical importance.

[B5] Harb M, Faris R, Gad A, Hafez O, Ramzy R, Buck A (1993). The resurgence of lymphatic filariasis in the Nile delta. Bulletin of World Health Organization.

[B6] Thompson D, Malone J, Harb M, Faris R, Huh O, Buck A (1996). Bancroftian filariasis distribution and diurnal temperature differences in the southern Nile delta. Emerging Infectious Diseases.

[B7] Subra R (1982). The distribution and frequency of *Culex pipiens quinquefasciatus *Say 1823 (Diptera, Culicidae) breeding places on the Kenya coast in relation to human sociological factors. Journal of Tropical Medicine and Hygiene.

[B8] Miller B, Nasci R, Godsey M, Savage H, Lutwama J, Lanciotti R (2000). First field evidence for natural vertical transmission of West Nile virus in *Culex univittatus *complex mosquitoes from Rift Valley province, Kenya. American Journal of Tropical Medicine and Hygiene.

[B9] Reiter P, Cordellier R, Ouma JO, Cropp CB, Savage HM, Sanders EJ, Marfin AA, Tukei PM, Agata NN, Gitau LG, Rapuoda BA, Gubler DJ (1998). First recorded outbreak of yellow fever in Kenya, 1992–1993. II. Entomologic investigations. American Journal of Tropical Medicine and Hygiene.

[B10] Kitron U (1998). Landscape ecology and epidemiology of vector-borne diseases: tools for spatial analysis. Journal of Medical Entomology.

[B11] Thomson MC, Connor SJ (2000). Environmental information systems for the control of arthropod vectors of disease. Medical and Veterinary Entomology.

[B12] Diuk-Wasser MA, Bagayoko M, Sogoba n, Dolo G, Toure MB, Traore SF, Taylor CE (2004). Mapping rice field anopheline breeding habitats in Mali, West Africa, using Landsat ETMz sensor data. International Journal of Remote Sensing.

[B13] Tennakoon SB, Murty VN (1992). Estimation of cropped area and grain yield of rice using remote sensing data. International Journal of Remote Sensing.

[B14] Okamoto K, Fukuhara M (1996). Estimation of paddy field area using the area *Anopheles *rice breeding mapping using the area ratio of categories in each mixel of Landsat TM. International Journal of Remote Sensing.

[B15] Singh VP, Singh AN (1996). A remote sensing and GIS-based methodology for the delineation and characterization of rainfed rice environments. International Journal of Remote Sensing.

[B16] Fang H, Wu B, Liu H, Huang X (1998). Using NOAA AVHRR and Landsat TM to estimate rice area year-by-year. International Journal of Remote Sensing.

[B17] Okamoto K, Yamakawa S, Kawashima H (1998). Estimation of flood damage to rice production in North Korea in 1995. International Journal of Remote Sensing.

[B18] Xiao X, Boles S, Frolking S, Salas W, Moore B, Li C (2002). Observation of flooding and rice transplanting of paddy rice fields at the site to landscape scales in China using vegetation sensor data. International Journal of Remote Sensing.

[B19] Wood B, Beck LR, Washino RK, Hibbard KA, Salute JS (1992). Estimating high mosquito-producing rice fields using spectral and spatial data. International Journal of Remote Sensing.

[B20] Hay SI, Packer MJ, Rogers DJ (1997). The impact of remote sensing on the study and control of invertebrate intermediate hosts and vectors of disease. International Journal of Remote Sensing.

[B21] Martin RDJ, Heilman JL (1986). Spectral reflectance patterns of flooded rice. Photogrammetric Engineering and Remote Sensing.

[B22] Barbosa PM, Casterad MA, Herrero J (1996). Performance of several Landsat 5 Thematic Mapper (TM) image classification methods for crop extent estimates in an irrigation district. International Journal of Remote Sensing.

[B23] Pope KO, Sheffner EJ, Linthicum KJ, Bailey CL, Logan TM, Kasischke ES, Birney K, Njogu AR, Roberts CR (1992). Identification of central Kenyan Rift Valley Fever virus vector habitats with landsat TM and evaluation of their flooding status with airborne imaging radar. Remote Sensing of the Environment.

[B24] Ryan R, Baldridge B, Schowedgerdt R, Choi T, Helder D, Blonski S (2003). IKONOS spatial resolution and image interpretability. Remote Sensing of Environment.

[B25] Service MW (1993). Mosquito ecology. Field sampling methods.

[B26] Wood B, Washino R, Beck L, Hibbard K, Pitcairn M, Roberts D, Rejmankova E, Paris J, Hacker C, Salute J, Sebesta P, Legters L (1991). Distinguishing high and low anopheline-producing rice fields using remote sensing and GIS technologies. Preventive Veterinary Medicine.

[B27] Grodecki J, Dial G (2002). IKONOS geometric accuracy validation. Proceeding of ISPRS commission I Mid-Term Symposium.

[B28] ERDAS (2005). ERDAS Software version 8.7 of ERDAS IMAGINE tour guide, field guide, and VirtualGIS.

[B29] ESRI ArcGIS 9.1 of arcMap, arcCatalog, arcToolbox and spatial analyst user's guide.

[B30] SPSS Inc.

[B31] Keating J, Macintyre K, Mbogo C, Githure J, Beier J (2004). Characterization of potential larval habitats for *Anopheles *mosquitoes in relation to urban land-use in Malindi, Kenya. International Journal of Health Geography.

[B32] Sithiprasasna R, Linthicum KJ, Liu GJ, Jones JW, Singhasivanon P (2003). Use of GIS-based spatial modeling approach to characterize the spatial patterns of malaria mosquito vector breeding habitats in northwestern Thailand. Southeast Asian J Trop Med Public Health.

[B33] Ishii T, Sohn S (1987). Highly polluted larval habitats of the *Culex pipiens *complex in central Sweden. American Journal Mosquito Control Association.

[B34] Lampman RL, Novak RJ (1996). Oviposition preferences of *Culex pipiens *and *Culex restuans *for infusion-baited traps. Journal of Am Mosq Control Assoc.

[B35] Webbe G (1961). Breeding sites of malaria vectors in relation to water management and agricultural practice in the Sukumaland area of Tanganyika. East African Medical Journal.

[B36] Mogi M, Sota T (1996). Physical and biological attributes of water channels utilized by *Culex pipiens pallens *immature in Saga City, southwest Japan. Journal of American Mosquito Control Association.

[B37] Jacob BG, Arheart KL, Griffith DA, Mbogo CM, Githeko AK, Regens JL, Githure JI, Novak R, Beier JC (2005). Evaluation of environmental data for identification of *Anopheles *(Diptera: Culicidae) aquatic larval habitats in Kisumu and Malindi, Kenya. Journal of Medical Entomology.

[B38] Jacob B, Regens J, Mbogo C, Githeko A, Keating J, Swalm C, Gunter J, Githure J, Beier J (2003). Occurrence and distribution of *Anopheles *(Diptera: Culicidae) larval habitats on land cover change sites in urban Kisumu and urban Malindi, Kenya. Journal of Medical Entomology.

[B39] Gu W, RJ Novak (2005). Habitat-based modeling of impacts of mosquito larval interventions on entomological inoculation rates, incidence, and prevalence of malaria. American Journal of Tropical Medicine and Hygiene.

[B40] Brengues J (1978). *Culex pipiens fatigans *Wiedemann in tropical Africa: its importance and its control. Medicina Tropical (Mars).

